# Supplementary material for: Unwrapping the Ciliary Coat: High‐Resolution Structure and Function of the Ciliary Glycocalyx
Source: Adv Sci (Weinh). 2025 Mar 5;12(16):2413355. doi: 10.1002/advs.202413355 (PMC12021028; doi:10.1002/advs.202413355)
Supplement: Supplementary file 1 — Supporting Information [file ADVS-12-2413355-s003.docx]

Supporting Information

Unwrapping the ciliary coat: high-resolution structure and function of the ciliary glycocalyx

Lara M. Hoepfner^1,a^, Adrian P. Nievergelt^2,4,a,^*, Fabrizio Matrino*^3^*, Martin Scholz^1^, Helen E. Foster*^3^*, Jonathan Rodenfels^2,5^, Alexander von Appen*^2,5^*, Michael Hippler^1,6,^*, Gaia Pigino^3,^*

^1^ *Institute of Plant Biology and Biotechnology, University of Münster, Schlossplatz 8,-48143 Münster, Germany*

*^2^ Max Planck Institute of Molecular Cell Biology and Genetics, Pfotenhauerstraße 108, 01307, Dresden, Germany*

*^3^ Human Technopole, V.le Rita Levi-Montalcini, 1, 20017, Milan, Italy*

*^4^ Lead contact*

*^5^ Cluster of Excellence Physics of Life, TU Dresden, 01062 Dresden, Germany*

*^6^ Institute of Plant Science and Resources, Okayama University, Kurashiki 710-0046, Japan*

** Corresponding authors: gaia.pigino@fht.org (G.P.), mhippler@uni-muenster.de (M.H), adrian@nievergelt-merz.ch (A.P.N.)*

*^a^ These authors contributed equally*


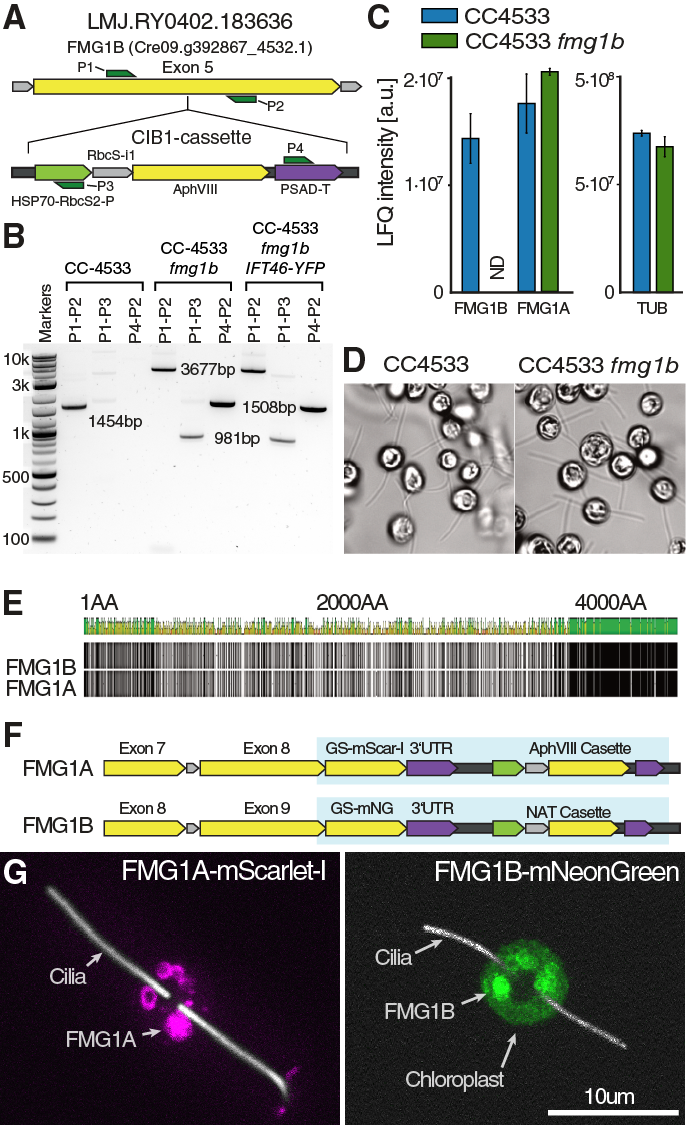


**Figure S1: Detailed genetic and proteomic analysis of FMG1A and FMG1B. A)** The CliP strain LMJ.RY0402.183636 (CC-4533 *fmg1b*) has a CIB cassette insertion in exon 5 of FMG1B. **B)** PCR analysis confirms insertion of the CIB cassette by sizing and specific amplification. **C)** LC-MS/MS analysis confirms absence of FMG1-B in CC-4533 *fmg1b*, while protein intensity for FMG1A is slightly but not significantly increased. Relative abundance of βTubulin in wildtype and mutant is shown as control. **D)** Both CC-4533 and CC-4533 *fmg1b* adhere to glass surfaces in stretched cilia conformation, indicative of gliding motility. **E)** Amino-acid alignment shows significant primary sequence divergence of most of the n-terminal part of both FMG1 isoforms and a conserved c-terminal region. **F)** Genomic regions of CRISPR/Cas mediated endogenous fluorescent knock-ins for both FMG1 isoforms. Exogenous insert region is highlighted in blue. **G)** Spinning-disk confocal z-projections showing similar intracellular localization of both FMG1 isoforms to large, ring-like or vesicle-like structures. Cilia overlaid in intensity-rescaled white color for reference. Scale bar is 10μm. Related to Figure 1.


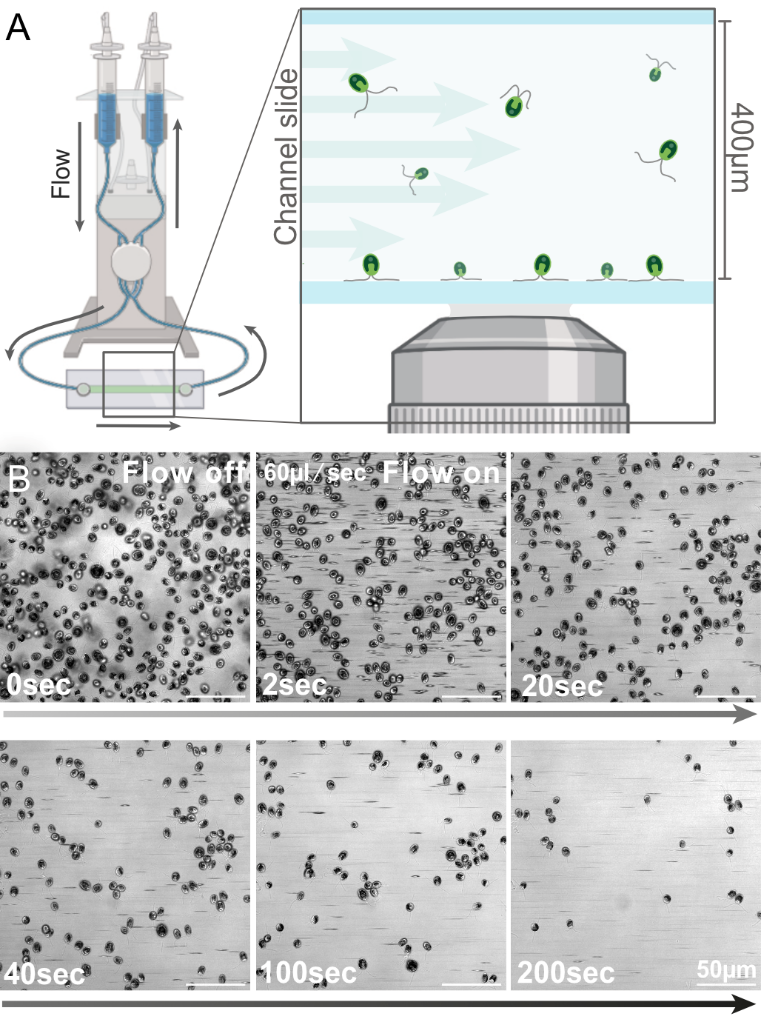


**Figure S2: Microfluidic measurement for adhesion strength of attached *C. reinhardtii* cells to a microscopy slide. A)** Schematic overview of the fluidic setup. Figure created with Biorender. Buffer is pumped from syringe reservoirs through a microscope slide with a microchannel by a peristaltic pump**. B)** Brightfield micrographs of adhered *C. reinhardtii* cells attached to the bottom polymer surface of the microchannel. Under flow, cells detach at a rate proportional to the strength of adhesion and can be seen moving as horizontal streaks through the image. Scale bar is 50μm. Related to Figure 1 and Figure 2.


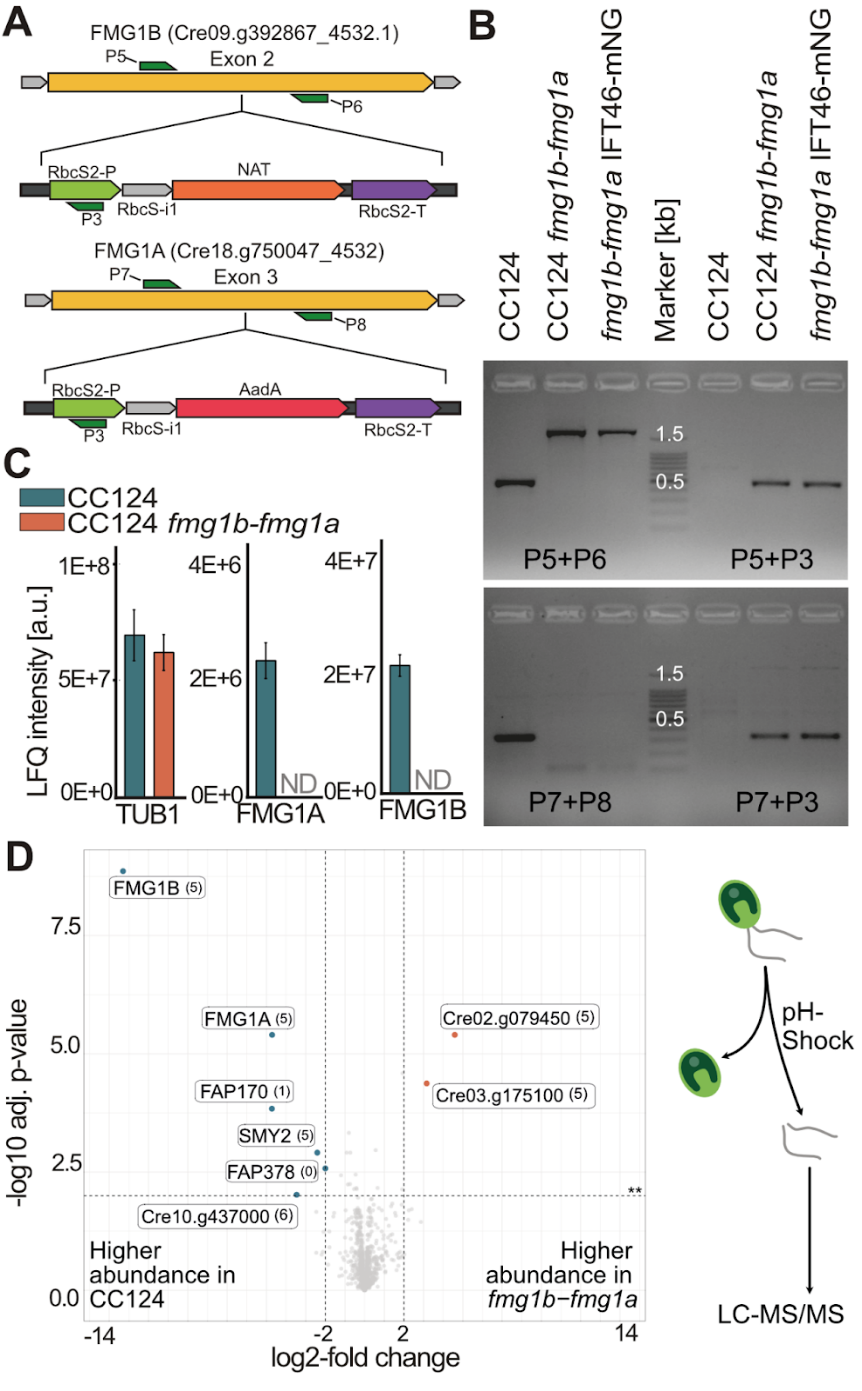


**Figure S3: Genetic and proteomic analysis of CC-124 *fmg1a fmg1b*. A)** Genomic maps of the glycocalyx deficient mutant, generated by CRISPR/Cas mediated insertion of NAT and AadA resistance cassettes in Exon2 and Exon3 of *fmg1b* and *fmg1a* respectively. **B)** PCR analysis confirming insertion of the cassette by sizing and specific amplification. **C)** LC-MS/MS analysis confirming the absence of FMG1B and FMG1A in CC-124 *fmg1a-fmg1b*. Relative abundance of βTubulin in wildtype and mutant is shown as control. **D)** Cilia proteome comparison of CC-124 and CC-124 *fmg1a-fmg1b* shows only minor changes in overall protein abundances. Bracketed numbers present replicates with imputed values (undetectable on peptide level). Data derives from five biological replicates. Related to Figure 2 and Figure 3.

| **AA** | **Motif** | **Prediction** | **EM**  **density** | **IS-CID**  **pep+HexNAc**  **CC-124** | **IS-CID**  **pep+HexNAc**  **Xu et al. 2020** | **HexNAc modified peptide** |
| --- | --- | --- | --- | --- | --- | --- |
| 49 | NVTT | ++ | + |  |  |  |
| 67 | NATK | + | + |  |  |  |
| 77 | NCTV | + | + |  |  |  |
| 107 | NQTT | + |  |  |  |  |
| 112 | NTTA | + |  |  |  |  |
| 162 | NMTI | + | + |  | + | LPLSSGNTMPDGYAT**NMT**ICGAAVQLLSAAGAVK |
| 263 | NASA | + |  |  |  |  |
| 305 | NGTT | + |  | + |  | SGTALPTALSACMAGPAVTAITATFA**NGT**TYSDGLTVTIK |
| 320 | NQTL | ++ | + |  |  |  |
| 406 | NNTA | + | + | + | + | SSPFSACALAS**NNT**ALVLTLASASTYTAGDIFNVK |
| 493 | NSTA | - |  |  | + | SVIIAAA**NST**AAK |
| 617 | NNTL | + |  |  |  |  |
| 623 | NMTA | + | + |  |  |  |
| 648 | NTTA | + |  |  |  |  |
| 693 | NGTV | + | + |  |  |  |
| 800 | NQSI | - | + |  |  |  |
| 819 | NCTL | + | + |  |  |  |
| 919 | NSSG | + | + | + | + | LSVAAAAVDGFSSSCNNVFTLF**NSS**GTAR |
| 1030 | NGTL | ++ | + |  |  |  |
| 1183 | NPTF | Prolin |  |  |  |  |
| 1243 | NTTS | - | + |  |  |  |
| 1367 | NNSD | + | + | + | + | LLG**NNSD**VYTGGDTFNFK |
| 1769 | NPTC | Proline |  |  |  |  |
| 3120 | NTSP | --- | + |  |  |  |
| 3633 | NTTK | +++ | + | + | + | ADCDAVFVFSGAG**NTT**K* |
| 3835 | NFTV | ++ |  |  |  |  |
| 3856 | NGTV | - | + |  | + | **NGT**VASCMLMPDR* |
| 3880 | NFSA | + | + | + | + | LLSAG**NFSA**GDTVNIKPEQAELR |
| 3925 | NLTS | + | + | + | + | LAAQVVNPALFANA**NLTS**ATAITVR* |
| 3963 | NVSS | + | + | + | + | **NVS**SCSLGADGVTLAVTIPAASFVGGDVLNIVPGQR* |
| 4029 | NATI | + |  |  |  |  |
| 4060 | NGSA | + |  | + |  | **NGS**AVASPLSACAVSADGLSLTLTAAATYKPMAGDTVDVAVSQTVLR |
| 4151 | NYSA | + |  |  |  |  |
| 4179 | NFTT | + |  |  | + | GLATGPLSCNVLIGDLTVTTTAG**NFTT**MASYAGK |
| 4260 | NASA | - |  |  |  |  |
| 4312 | NTSV | - |  | + | + | FFDGL**NTS**VAGR |
| 4345 | NTTD | - |  |  |  |  |
| 4357 | NVTY | + |  |  |  |  |
| 4366 | NATS | - |  |  |  |  |

**Table S1: List of N-glycosites of FMG1B identified by computational and experimental methods.** Net*N*-glyc predicted *N*-glycosites in FMG1B correlate greatly with additional densities observed in CryoEM, IS-CID-MS/MS identified glycopeptides in CC-124 and identified glycopeptides for FMG1B in ^2^. Peptides marked with a * are indistinguishable from FMG1A. Related to Figure 3.

|  | **TopN without IS-CID**  **(Cilia and whole cell proteomes)** | **IS- CID**  **(Glyco**  **proteomics)** | **TopN without IS-CID**  **(Disulfide identification )** | |  |
| --- | --- | --- | --- | --- | --- |
|  | 240min | 240min | 240min | 180min |  |
| **Eluent composition** | Peptide trapping: 0.05% trifluoroacetic acid (TFA) in ultrapure water (A1), 0.05% TFA in 80% acetonitrile (B1)  Peptide separation: 0.1% formic acid (FA) in ultrapure water (A2), 0.1% FA in 80% acetonitrile (B2) | | | | **LC parameters** |
| **Trap Column** | C18 PepMap 100, 300 µm x 5 mm, 5 µm particle size, 100 Å pore size; Thermo Scientific | | | |  |
| **Peptide trapping** | 2.5% B1 at 5 μL/min for 5 min | | | |  |
| **Flow rate** | 300nl/min | | | |  |
| **Separation column** | Acclaim PepMap C18, 75 µm x 50 cm, 2 µm particle size, 100 Å pore size; Thermo Scientific | | | |  |
| **Gradient for peptide separation** | 2.5-5% B2 over 5min,  5-17.5% B2 over 50min,  17.5-40% B2 over 100min,  40-99% B2 over 10min, 99% B2 over 20min,  99-2.5% B2 over 5min,  2.5% B2 over 50min | | | 2.5-5% B2 over 5min  5–40%B2 over 90 min,  45–99 % B2 over 10 min  99% B2 for 20 min  99–2.5% over 5 min  2.5% for 50 min |  |
| **In-source CID** | off | 80eV | off | | **MS1 settings** |
| **Use lock mass** | off | | | |  |
| **Ion mode** | positive | | | |  |
| **Resolution at m/z 200 (FWHM)** | 70,000 | | | |  |
| **Chromatographic peak width** | 15sec | | | |  |
| **AGC target** | 3e6 | | | |  |
| **Max. injection time** | 50ms | | | |  |
| **Scan range** | 350-1400m/z | 600-3000m/z | 400-1600m/z | |  |
| **Mass tag** | off | on (±203.07937, ±101.53969, ±67.69312, ±50.76984) | off | |  |
| **TopN** | 12 | | | | **MS2 settings** |
| **Resolution at m/z 200 (FWHM)** | 17500 | | 35000 | |  |
| **Isolation window** | 1.5m/z | | | |  |
| **AGC target** | 5e4 | | | |  |
| **Max injection time** | 80sec | 120ms | 120ms | |  |
| **NCE** | 27 | 30 | 28 | |  |
| **Min. AGC target** | 8e2 | 1.25e3 | 1.25e3 | |  |
| **Intensity threshold** | 1e4 | | | |  |
| **Charge exclusion** | unassigned,1,5-8,>8 | unassigned,1,5-8,>8 | unassigned, 1,2,6 – 8, >8 | |  |
| **Dynamic exclusion** | 60s | | | 45s |  |

**Table S2:** Basic LC and MS/MS parameter employed for data-dependent acquisition. Related to methods.
